# Stable Isotope Ratio and Elemental Profile Combined with Support Vector Machine for Provenance Discrimination of Oolong Tea (Wuyi-Rock Tea)

**DOI:** 10.1155/2017/5454231

**Published:** 2017-04-03

**Authors:** Yun-xiao Lou, Xian-shu Fu, Xiao-ping Yu, Zi-hong Ye, Hai-feng Cui, Ya-fen Zhang

**Affiliations:** Zhejiang Provincial Key Laboratory of Biometrology and Inspection & Quarantine, College of Life Sciences, China Jiliang University, Hangzhou 310018, China

## Abstract

This paper focused on an effective method to discriminate the geographical origin of Wuyi-Rock tea by the stable isotope ratio (SIR) and metallic element profiling (MEP) combined with support vector machine (SVM) analysis. Wuyi-Rock tea (*n* = 99) collected from nine producing areas and non-Wuyi-Rock tea (*n* = 33) from eleven nonproducing areas were analysed for SIR and MEP by established methods. The SVM model based on coupled data produced the best prediction accuracy (0.9773). This prediction shows that instrumental methods combined with a classification model can provide an effective and stable tool for provenance discrimination. Moreover, every feature variable in stable isotope and metallic element data was ranked by its contribution to the model. The results show that *δ*^2^H, *δ*^18^O, Cs, Cu, Ca, and Rb contents are significant indications for provenance discrimination and not all of the metallic elements improve the prediction accuracy of the SVM model.

## 1. Introduction

Oolong tea is a traditional beverage favoured by consumers all over the world for its pleasurable aroma and taste. In addition, Oolong tea is a rich source of antioxidants, such as tea polyphenol and tea polysaccharide, so it is also reported as a functional drink that combats obesity, hypoglycaemia, and oral bacterial infection [1–4].

For tea products, their aroma and savor are influenced by many aspects, such as geographical origin [5, 6], tea specie, cultivation, and processing method [7, 8]. Among these aspects, the geographical and natural conditions in which the tea trees grow are widely perceived to be a key factor. Therefore, in China, the majority of famous teas are named for their provenance, such as the Anxi-Tieguanyin tea, the West Lake-Longjing tea, and the Anji-White tea.

Wuyi-Rock tea is originally cultivated in a mountain in the north of Fujian Province (Wuyi Mountain). Contributed to by the unique climate and edatope of Wuyi Mountain, Wuyi-Rock tea (WRT) is recognized as one of the most prestigious Oolong teas for its special savor and long-lasting fragrance. Therefore, Wuyi-Rock tea has been awarded a protected geographical indication (PGI) and exported to more than 30 countries. However, the actual yield of WRT is limited, and it cannot satisfy the needs of consumers. In various markets, many teas labeled as Wuyi-Rock tea were actually cultivated outside the protected production area; some of them are not even cultivated in Fujian Province. Although the taste and aroma of non-Wuyi-Rock teas (NWRT) are inferior to authentic WRT, teas planted in different geographical origins still have a similar appearance, and they can hardly be distinguished just by the naked eye. In traditional sensory analysis, WRT was tasted by professional tea tasters, and then counterfeits were identified based on a series of sensory scores. However, the result of sensory analysis depends a great deal on subjective decisions by tea tasters, and a well-trained tea taster is hard to find. Therefore, an urgent demand exists for developing a more effective and stable technique to discriminate the provenances of tea products.

In recent years, analytical methods based on instrumental technology have been widely applied in food quality control [9, 10]. Isotope ratio mass spectrometry (IRMS) is a technique for measuring the isotope content, which is a highly indicative parameter in provenance discrimination. For example, the stable isotope ratio of hydrogen (*δ*^2^H) in plants is influenced by the latitude and altitude of the production site. The concentration of deuterium in the water decreases when clouds form above the ocean. Then, as rainwater falls and the clouds move inland and gain altitude, the content of *δ*^2^H in rainwater decreases gradually [11]. As a result, an isotopic gradient exists in groundwater from coast to inland. Moreover, the variation of oxygen-18 (*δ*^18^O) follows the same pattern as hydrogen in the hydrosphere [12]. The isotope ratio of carbon (*δ*^13^C) is strongly environmentally dependent; plants cultivated in humid environments have a lower *δ*^13^C than plants in arid environments. The isotope ratio of nitrogen (*δ*^15^N) is influenced by agricultural practices; plants treated with organic fertilizer develop a higher *δ*^15^N content than plants treated with chemical fertilizer. For provenance analysis, IRMS has been successfully used for the analysis of orange juice, fruits, cow milk, and wine [13–16]. In addition to isotopes, trace element profiling is also available. There are various metallic elements in agricultural food; some of them are easily affected by edaphic and environmental factors, such as fertilization, soil type, climate, and temperature. Both inductively coupled plasma mass spectrometry (ICP-MS) and atomic spectroscopy are tools for quantitative determination of trace elements. In provenance discrimination, element profiling has been used for honey, onion, black tea, and wine [17–21].

This paper aims to develop an automatic analytical method for discriminating geographical origin of WRT by stable isotope and trace element contents. To model the complicated relationship between the measured data and production site, nonlinear multivariate classification models are usually used [22–25]. Compared with other nonlinear models such as kernel partial least squares (PLS) and artificial neural networks (ANNs), support vector machine (SVM) analysis is more suitable for the target data in this experiment because of the small sample size [26–28]. Consequently, the classification model was built based on SVM. Then, each variable of isotope and element data was ranked by its contribution to the model.

## 2. Materials and Methods

### 2.1. Tea Samples

**Figure 1:**
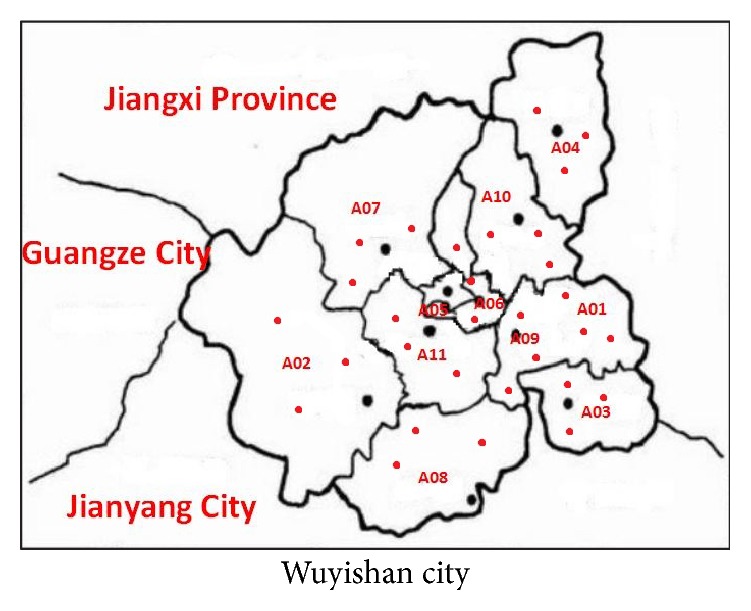
Tea samples collected in Wuyishan.

In total, 99 authentic WRT samples were collected from 11 main rich-producing areas in Wuyishan, and 33 NWRT samples were collected from 11 different production sites. All of the samples were made of spring teas picked in 2015. Before analysis, the samples were preserved in cold and dry storage with lightproof packaging. Detailed information about the above samples is displayed in Table 1 and Figure 1.

The origin of Wuyi-Rock tea is the administrative area of Wuyishan city, and Wuyishan has 11 subdistricts. The range of sample collection covered all over the whole Wuyishan city area. So the number of samples is adequate. All the collected tea samples were obtained directly from the local tea processing space with the help of official department. All the tea samples belonged to “Wuyi-Rock tea,” and the non-Wuyi-Rock tea samples were purchased outside the protected production area such as Jianyang, Jianou, and Ganzhou.

### 2.2. Isotopic Ratio Determinations

*δ* ^13^C, *δ*^15^N, *δ*^18^O, and *δ*^2^H were measured using a MAT-253 isotope ratio mass spectrometer (Thermo Fisher, USA) connected to a Flash-2000 organic elemental analyser (Thermo Fisher, USA). During carbon and nitrogen isotope analysis, the quartz tube of the reactor was packed with chromium trioxide, high purity copper, and silver cobalt oxide to completely oxidize the organic matter. The carbon and nitrogen element carried by helium gas entered the IRMS in the form of CO_2_ and N_2_, respectively. The standard CO_2_ and N_2_ gases were used as reference gas before and after the isotope test of organic matter, and the detection of the instrument state in the sample analysis was completed by the standard sample such as labeled urea, IAEA-600, IAEA-CH-3, and VPDB (Vienna Pee Dee Belemnite). Similarly, the instrument state was detected by benzoic acid, IAEA-601, IAEA-602, IAEA-CH-7, and VSMOW (Vienna Standard Mean Ocean Water) in the analysis process of hydrogen and oxygen isotope. Each sample was repeated three times.

The measured values (^13^C/^12^C, ^15^N/^14^N, ^18^O/^16^O, and ^2^H/^1^H) are usually presented as isotopic deviations, *δ*, defined as follows:

(1) $$\begin{array}{c} \delta \text{‰}=\left(\;\frac{R_{s}}{R_{std}}-1\;\right)\times 1000, \end{array}$$

where *R*_*s*_ is the measured value of the sample and *R*_std_ is the measured value of an international standard. *R*_*s*_ and *R*_std_ are the ratios of the heavier isotope and lighter isotope of an element.

In isotope profiling, calibration was conducted according to calibrated-urea and calibrated-benzoic acid standards as well as the IAEA-600, IAEA-601, IAEA-602, IAEA-CH-3, and IAEA-CH-3 standards of the International Atomic Energy Agency (IAEA, Vienna).

### 2.3. Metal Determinations

Before metallic element detection, all of the tea samples were pretreated with microwave assisted digestion. The samples were dried before digestion process (placed in the oven for 4 hours at 80°C). All of the tea samples were manufactured in May 2015 and simultaneously analysed. Water content of tea samples was less than 6% before drying. 0.3 g of each dried tea sample was placed into a digestion vessel. Then, 1 mL ultrapure water and 5 mL nitric acid were added to the vessel. Finally, the digestion vessel was heated in the microwave cavity (2450 MHz). The vibration of gas pressure in the digestion procedure was conducted as follows: (1) ramping from normal pressure to 0.5 Mpa and holding at 0.5 Mpa for 70 s, (2) ramping to 1.0 Mpa for 50 s, (3) ramping to 1.5 Mpa for 50 s, and (4) ramping to 2.0 Mpa for 300 s. After the digestion, we evaporated the excess nitric acid in 130°C. When the temperature of the digestion vessel had cooled to room temperature, the digested liquid was moved to a volumetric flask and diluted to 50 mL with ultrapure water.

Subsequently, concentrations of 14 metallic elements were measured. The concentrations of Ti, Cr, Co, Ni, Cu, Zn, Rb, Cd, Cs, Ba, and Sr were detected using an X Series-IIICP-MS (Thermo Fisher, The USA). The information about the working condition and parameters for ICP-MS is shown in Table 2. The concentrations of Ca, Mg, and Mn were analysed by HITACHI 180-50 flame atomic absorption spectroscopy (FAAS, HITACHI, Japan). The main parameters for FAAS are presented in Table 3. The results of ICP-MS and FAAS were calibrated by mixed standard solution (GSB04-1767-2004) and biological component analysis standard substance, tea (GBW10052), respectively.

### 2.4. Data Splicing

All the data analysis was performed using MATLAB 7.14.0.739 (Mathworks, Sherborn, MA). For data splicing, the data of IRMS can be described as an *n* × *p* matrix *A* with *n* rows and *p* columns. *n* represents the number of samples and *p* is the number of features in this paper (*n* = 132, *p* = 4). In the same way, an *n* × *q* matrix *B* (in this paper, *q* = 14) was obtained from metallic element detection. Then, the columns of matrix *B* were arranged behind the last column of matrix *A*. As a result, a union matrix *C* (with *n* rows and *p* + *q* columns) was formed that contains both the isotope and metallic element information.

Before data analysis, each variable in matrix *C* was normalized as follows:

(2) $$\begin{array}{c} x^{*}=\frac{x-x_{min}}{x_{max}-x_{min}}, \end{array}$$

where *x* is the value of *i*th (*i* = 1 : 132) row and* j*th (*j* = 1 : 18) column, *x*_max_ is the max value in the* j*th column, and *x*_min_ is the min value in the* j*th column.

### 2.5. SVM Analysis

The support vector machine algorithm is a type of classification and regression model for supervised machine learning. The kernel function is the main factor in the SVM algorithm. Kernels have the advantage of operating in the input space, where the solution of the classification problem is a weighted sum of kernel functions evaluated at the support vectors. SVM is designed to find an optimal plane that all the sample units can be divided into two classes in a multidimensional space. The optimal plane is in the middle of the nearest points between two classes and makes the distance as far as possible. For *N* variables, the optimal hyperplane is of *N*-1 dimensions [29].

After the data splicing was performed, three SVM classification models were established, based on the isotope data (Matrix *A*), the metallic element data (Matrix *B*), and the union data (Matrix *C*). For all of the 132 samples (99 WRT samples and 33 NWRT samples), 88 of them (including WRT and NWRT samples) were selected as a training class at random and the other 44 were put into a prediction class.

To estimate the performance of the SVM model, the sensitivity and specificity were calculated as follows:

(3) Sens.=TPTP+FN,Spec.=TNTN+FP,

where TP and FN represent the number of true positives and false negatives, respectively, and TN and FP denote the number of true negatives and false positives, respectively.

### 2.6. Variable Ranking

For SVM models, it is obvious that each variable does not contribute equally to prediction accuracy. In addition, some useless information may even have negative influence on prediction, so the significance of each measured isotope and element was investigated in this paper. For each feature, the column was removed from the data matrix and a new SVM model based on the incomplete data was built. In this way, each feature of the isotope and element data was separately removed, and 18 models were built. Then, the models were compared, and if a model showed a lower accuracy, the missing feature was considered important in provenance discrimination. Using this method, every variable was ranked by its contribution.

## 3. Results and Discussion

*δ* ^13^C in plant samples is mainly affected by the metabolic pathway of plant photosynthesis, so *δ*^13^C is significantly different between different plants, while *δ*^15^N is mainly under the influence of such regional agricultural activities as fertilization [12]. *δ*^2^H and *δ*^18^O are affected by atmospheric water cycle. Obviously, they have dimensional and land effects through the meteorological cycle of evaporation, condensation, and precipitation. Decreasing temperatures causes a progressive heavy-isotope depletion of the precipitation when the water vapour from oceans in equatorial regions moves to higher latitudes and altitudes [11]. *δ*^2^H and *δ*^18^O in plants are affected by *δ*^2^H and *δ*^18^O in the surrounding environment, so they are well used to characterize the origin of agricultural products [12]. The characteristics of metallic elements in plants are not only related to the composition of mineral elements in the soil, but also affected by varieties, climate, and agricultural activities [30, 31]. Alkaline metals, especially Cs and Rb being easily mobilised in the soil and transported into plants, are good indicators of geographical identity [12]. Cu, Zn, and Cd in the soil will be affected by agricultural activities (organic fertilizer), so these elements in plants will also be affected [32–34]. In conclusion, it is necessary to evaluate the influence of these trace elements in the identification of tea samples.

The results of isotope and metallic element profiling are presented in Tables 4 and 5. The tables demonstrate a considerable difference in isotope and metallic content, but distinguishing the provenance of the sample just by these values proved difficult. Chemometric models are powerful tools in such situations.

Three SVM models based on isotope data, element content data, and coupled data were established, and their prediction results are shown in Table 6. The accuracy of isotope-SVM model reached 0.9318, and only 3 samples were mispredicted. Although the performance of element-SVM models (0.7727) was not very satisfactory, when it was applied coupled with isotope data, the model can greatly improve predictions, and the accuracy of the coupled model reached 0.9773.

**Figure 2:**
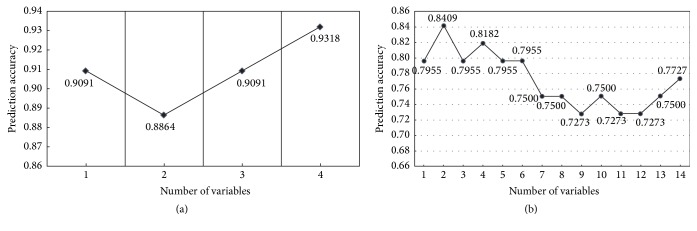
(a) Feature selection of isotope and prediction accuracy. (b) Feature selection of metallic element and prediction accuracy.

The rank of each feature is reported in Table 7. In the table, *δ*^2^H, *δ*^18^O, Cs, Cu, Ca, and Rb contents are ranked highest, so they are the most significant indication in provenance analysis of WRT. Moreover, in further analysis, each feature was accumulatively assembled by its rank order and the variation of accuracy was plotted in Figure 2 when a new variable was added. As shown in Figure 2, the prediction accuracy was reduced as the *δ*^18^O feature was added. This result may be caused by the overlapping chemical information between *δ*^2^H and *δ*^18^O because they have analogous variation in the hydrosphere. Therefore, the relationship between *δ*^2^H and *δ*^18^O was examined, and the correlation coefficient between *δ*^2^H and *δ*^18^O reached 0.8634, which strongly supports this assumption. Afterwards, the model achieved better performance as the variables of *δ*^15^N and *δ*^13^C were added. In element profiling, the SVM model achieved the best prediction result when only Cs and Cu contents were applied. The prediction accuracy decreased as more element features were used, so some metal elements were not significant in the identification of Wuyi-Rock tea geographical origin.

## 4. Conclusion

The origin place of Wuyi-Rock tea is the typical Danxia landform constituting purple soil, red soil, and moist sandy soil and the microenvironment is unique and exclusive. According to the results of tea identification, the importance of *δ*^2^H and *δ*^18^O exactly reflects the particular climatic environment in Wuyishan. *δ*^13^C mainly reflects the difference between different plants, and *δ*^15^N is easily influenced by the agricultural activities [12]; therefore, the importance of *δ*^13^C and *δ*^15^N is not significant in the identification. Cs, Rb, and Sr have a higher contribution to Wuyi-Rock tea discrimination than great majority of elements, which has illustrated that the special geology in Wuyishan area provides unique features of trace elements for Wuyi-Rock tea. The contents of Cu, Ca, and Zn in Wuyi-Rock tea were affected by many factors, such as the kind of soil, fertilization, and tea varieties, so it needed further investigation and analysis of the relationship between identification and those aspects mentioned above in Wuyishan tea field.

In this paper, isotope and metallic element analyses demonstrate the potential for geographical origin discrimination of Wuyi-Rock tea. As a nonlinear model, SVM was performed for classification, and the chemical information of isotopes and metallic elements is complementary in provenance discrimination. In addition, the ranks of isotope and element features were carried out using established methods. The result shows that *δ*^2^H, *δ*^18^O, Cs, Cu, Ca, and Rb contents are significant in provenance analysis, *δ*^2^H and *δ*^18^O are interrelated, and not every element is helpful in geographical origin discrimination.

## Figures and Tables

**Table 1:** Detailed information of the samples.

Number	Producing area	Size of sample^a^	Type^b^
A01	Shangmei, Wuyi, Fujian Province	9	A
A02	Xingcun, Wuyi, Fujian Province	9	A
A03	Wufu, Wuyi, Fujian Province	9	A
A04	Langu, Wuyi, Fujian Province	9	A
A05	Chongan Street, Wuyi, Fujian Province	9	A
A06	Xinfeng Street, Wuyi, Fujian Province	9	A
A07	Yangzhuang, Wuyi, Fujian Province	9	A
A08	Xingtian, Wuyi, Fujian Province	9	A
A09	Xiamei, Wuyi, Fujian Province	9	A
A10	Wutun, Wuyi, Fujian Province	9	A
A11	Wuyi Street, Wuyi, Fujian Province	9	A
N01	Jianyang, Fujian Province	3	N
N02	Jianou, Fujian Province	3	N
N03	Zhangzhou A, Fujian Province	3	N
N04	Zhangzhou B, Fujian Province	3	N
N05	Quanzhou, Fujian Province	3	N
N06	Guangxi Province	3	N
N07	Guizhou Province	3	N
N08	Ganzhou, Jiangxi Province	3	N
N09	Wuyuan, Jiangxi Province	3	N
N10	Songxi, Fujian Province	3	N
N11	Zhenghe, Fujian Province	3	N

^a^The number of the tea samples from the production site.

^b^A: Wuyi-Rock tea; N: non-Wuyi-Rock tea.

**Table 2:** Optimized instrumental conditions and parameters of the ICP-MS.

Parameter	Setting value
Forward power	1200 W
Scanning times	100 times
Scanning mode	Peak height
Dwell time	10 ms
Acquisition time	20 s
Sample uptake rate	1 mL/min
Coolant gas flow rate	14 L/min
Auxiliary gas flow rate	0.75 L/min
Nebulizer gas flow rate	0.92 L/min

**Table 3:** Main measurement parameters for Ca, Mg, and Mn.

Element	Wavelength	Bandpass	Ethyne gas flow rate	Air flow rate
Ca	422.7 nm	2.6 nm	2.6 L/min	9.4 L/min
Mg	285.2 nm	2.6 nm	2 L/min	9.4 L/min
Mn	279.5 nm	2.3 nm	2.3 L/min	9.4 L/min

**Table 4:** Results of provenance experiment: averages values of 4 stable isotopes in WRT and NWRT samples.

Number	*δ* ^13^C	*δ* ^2^H	*δ* ^18^O	*δ* ^15^N
A01	−26.77 ± 0.68	−80.90 ± 9.90	24.44 ± 0.33	1.14 ± 1.17
A02	−27.90 ± 0.76	−74.85 ± 7.54	23.39 ± 2.49	2.26 ± 0.93
A03	−27.14 ± 0.50	−77.05 ± 8.27	23.01 ± 0.81	2.55 ± 1.54
A04	−27.78 ± 0.96	−86.32 ± 0.83	22.18 ± 1.11	1.71 ± 1.06
A05	−26.79 ± 0.67	−78.34 ± 4.39	23.96 ± 1.49	2.76 ± 0.88
A06	−27.95 ± 0.78	−72.42 ± 5.61	25.05 ± 0.37	1.06 ± 2.46
A07	−27.56 ± 1.61	−86.27 ± 12.88	21.65 ± 2.63	1.40 ± 1.38
A08	−26.97 ± 0.56	−78.89 ± 4.15	24.24 ± 0.72	4.74 ± 2.10
A09	−27.50 ± 0.33	−86.99 ± 14.94	23.36 ± 3.10	2.54 ± 1.49
A10	−27.18 ± 0.12	−89.79 ± 6.63	24.56 ± 1.57	3.71 ± 0.96
A11	−26.90 ± 0.21	−73.76 ± 2.04	24.41 ± 0.81	1.83 ± 0.52
N01	−28.56 ± 0.19	−102.59 ± 0.89	18.63 ± 0.17	6.12 ± 0.22
N02	−26.96 ± 0.08	−89.39 ± 2.34	20.41 ± 0.44	3.63 ± 0.03
N03	−27.77 ± 0.07	−111.98 ± 1.55	18.34 ± 0.16	7.06 ± 0.22
N04	−28.34 ± 0.08	−111.98 ± 0.78	18.24 ± 0.22	6.78 ± 0.10
N05	−27.29 ± 0.15	−98.31 ± 1.83	22.39 ± 0.39	4.19 ± 0.07
N06	−27.84 ± 0.06	−107.15 ± 1.60	18.71 ± 0.25	4.14 ± 0.02
N07	−26.80 ± 0.09	−112.65 ± 0.64	16.65 ± 0.30	6.50 ± 0.07
N08	−28.04 ± 0.16	−103.89 ± 2.10	23.24 ± 0.20	1.98 ± 0.24
N09	−28.35 ± 0.11	−107.52 ± 1.83	18.48 ± 0.02	0.79 ± 0.02
N10	−27.88 ± 0.13	−107.75 ± 1.03	19.11 ± 0.35	4.91 ± 0.24
N11	−26.85 ± 0.14	−102.27 ± 1.97	21.09 ± 0.59	1.30 ± 0.18

**Table 5:** Results of provenance experiment: averages values of 14 element concentrations (*μ*g/g) in WRT and NWRT samples.

Number	Ti	Cr	Co	Ni	Cu	Zn	Rb
A01	27.56 ± 1.25	5.31 ± 1.05	0.88 ± 0.28	12.58 ± 2.61	11.65 ± 0.95	30.56 ± 1.17	50.89 ± 10.16
A02	27.06 ± 1.07	5.43 ± 0.26	0.26 ± 0.17	10.19 ± 1.22	11.40 ± 0.12	29.61 ± 3.51	40.11 ± 6.92
A03	25.78 ± 1.39	5.04 ± 0.65	0.14 ± 0.02	9.68 ± 1.28	10.08 ± 0.72	27.67 ± 3.66	46.22 ± 15.16
A04	23.72 ± 2.74	5.54 ± 0.16	0.27 ± 0.08	12.61 ± 1.17	15.78 ± 1.77	29.67 ± 3.18	66.22 ± 22.27
A05	27.06 ± 3.82	5.29 ± 0.71	0.24 ± 0.02	10.58 ± 0.61	10.54 ± 1.89	33.11 ± 9.73	35.72 ± 15.54
A06	28.22 ± 5.55	4.99 ± 0.18	0.48 ± 0.15	10.09 ± 0.83	11.20 ± 1.60	32.28 ± 2.60	59.39 ± 18.63
A07	24.39 ± 2.75	5.01 ± 0.59	0.33 ± 0.08	11.63 ± 2.02	12.92 ± 1.22	27.83 ± 7.55	69.08 ± 17.80
A08	24.67 ± 5.80	4.62 ± 0.29	0.41 ± 0.36	10.68 ± 2.50	12.10 ± 1.61	28.11 ± 9.48	80.78 ± 35.31
A09	26.22 ± 3.91	4.07 ± 0.95	0.30 ± 0.18	9.04 ± 2.24	11.38 ± 0.66	30.94 ± 6.46	40.11 ± 19.16
A10	26.94 ± 0.67	4.89 ± 0.27	0.88 ± 0.17	11.56 ± 1.57	13.68 ± 0.72	21.06 ± 2.51	54.94 ± 6.42
A11	32.06 ± 2.84	4.68 ± 0.40	0.28 ± 0.04	10.52 ± 0.20	12.06 ± 0.69	52.17 ± 27.66	53.11 ± 17.23
N01	20.33 ± 0.50	3.88 ± 0.21	0.17 ± 0.003	9.16 ± 0.28	10.39 ± 0.65	19.17 ± 0.29	43.17 ± 0.93
N02	28.94 ± 0.25	5.17 ± 0.08	0.93 ± 0.01	14.23 ± 0.34	13.39 ± 0.31	40.28 ± 1.02	104.33 ± 0.67
N03	24.06 ± 0.48	4.89 ± 0.09	0.19 ± 0.02	10.79 ± 0.24	11.67 ± 0.14	18.00 ± 0.58	73.83 ± 0.76
N04	20.78 ± 0.42	4.63 ± 0.12	0.22 ± 0	9.73 ± 0.32	11.69 ± 0.07	18.33 ± 1.01	69.22 ± 0.10
N05	22.72 ± 0.51	4.94 ± 0.09	0.24 ± 0.01	10.08 ± 0.48	11.86 ± 0.67	19.39 ± 0.98	45.11 ± 0.79
N06	31.17 ± 2.90	4.50 ± 0.07	0.64 ± 0.02	11.75 ± 0.17	15.68 ± 0.18	30.33 ± 2.17	347.78 ± 5.85
N07	39.61 ± 1.70	4.26 ± 0.02	1.35 ± 0.03	17.44 ± 0.25	23.67 ± 0.17	36.72 ± 2.12	59.17 ± 0.88
N08	21.17 ± 0.44	4.74 ± 0.08	0.48 ± 0.02	11.89 ± 0.79	30.39 ± 0.54	35.33 ± 8.99	117.17 ± 1.36
N09	29.72 ± 0.92	4.86 ± 0.06	0.24 ± 0.01	16.34 ± 0.18	27.89 ± 0.42	33.06 ± 0.35	76.56 ± 0.69
N10	21.06 ± 0.86	4.62 ± 0.08	0.28 ± 0.01	9.59 ± 0.29	13.36 ± 0.32	18.56 ± 1.34	78.83 ± 1.32
N11	29.17 ± 1.17	4.28 ± 0.09	0.34 ± 0.02	10.31 ± 0.26	15.85 ± 0.37	33.50 ± 0.44	92.56 ± 2.18
Ref	27.50	4.42	0.42	13.62	34.00	30.17	60.50

LOD (ng/L)	0.0019	0.025	0.001	0.008	0.045	0.091	0.001

Number	Cd	Cs	Ba	Sr	Ca	Mg	Mn

A01	0.07 ± 0.01	0.53 ± 0.26	14.24 ± 1.53	15.56 ± 1.92	3572.22 ± 233.53	2244.44 ± 133.68	1500.00 ± 317.54
A02	0.07 ± 0.01	0.48 ± 0.14	10.64 ± 2.63	15.00 ± 2.89	4122.22 ± 296.43	2266.67 ± 109.29	888.89 ± 350.13
A03	0.04 ± 0.01	0.44 ± 0.18	12.09 ± 4.20	15.56 ± 1.92	4288.89 ± 211.04	2305.56 ± 250.19	350.00 ± 152.75
A04	0.07 ± 0.01	0.32 ± 0.10	32.78 ± 13.76	13.33 ± 2.89	4027.78 ± 511.08	2633.33 ± 217.94	1038.89 ± 330.96
A05	0.08 ± 0.06	0.26 ± 0.19	15.01 ± 13.13	9.44 ± 0.96	3816.67 ± 464.58	2333.33 ± 389.80	966.67 ± 327.87
A06	0.06 ± 0.02	0.34 ± 0.03	7.68 ± 0.34	13.33 ± 3.33	3711.11 ± 475.61	2161.11 ± 110.97	872.22 ± 227.51
A07	0.10 ± 0.07	1.82 ± 1.22	22.17 ± 2.89	13.89 ± 2.54	4027.78 ± 500.09	2277.78 ± 409.72	1088.89 ± 460.17
A08	0.06 ± 0.05	1.26 ± 0.09	13.38 ± 3.94	12.78 ± 3.47	3872.22 ± 453.79	2127.78 ± 437.90	1194.44 ± 395.23
A09	0.09 ± 0.04	0.57 ± 0.28	8.10 ± 0.98	12.22 ± 3.85	3661.11 ± 431.51	2072.22 ± 58.53	905.56 ± 194.60
A10	0.04 ± 0.02	0.86 ± 0.48	12.04 ± 2.37	12.78 ± 3.47	3466.67 ± 180.28	2427.78 ± 122.85	1588.89 ± 242.86
A11	0.04 ± 0.01	0.39 ± 0.21	12.87 ± 7.39	12.78 ± 3.47	3200.00 ± 251.66	2327.78 ± 48.11	694.44 ± 327.59
N01	0.03 ± 0.01	1.08 ± 0.04	18.78 ± 0.79	15.00 ± 2.89	3666.67 ± 196.50	2227.78 ± 25.46	827.78 ± 82.21
N02	0.09 ± 0.01	2.406 ± 0.03	12.46 ± 0.22	12.22 ± 3.85	3605.56 ± 9.62	2294.44 ± 9.62	1394.44 ± 25.46
N03	0.04 ± 0.01	1.58 ± 0.02	16.83 ± 0.17	15.00 ± 2.89	3450.00 ± 196.50	2022.22 ± 75.15	650.00 ± 33.33
N04	0.04 ± 0.01	1.73 ± 0.01	22.11 ± 0.92	13.89 ± 2.54	3600.00 ± 50.00	1950.00 ± 16.67	1161.11 ± 34.69
N05	0.04 ± 0.01	1.64 ± 0.02	8.04 ± 0.12	14.44 ± 3.85	3205.56 ± 110.97	1972.22 ± 85.53	727.78 ± 19.24
N06	0.06 ± 0.02	2.80 ± 0.07	12.59 ± 0.38	15.56 ± 1.92	2688.89 ± 25.46	2183.33 ± 28.87	788.89 ± 9.62
N07	0.07 ± 0.01	1.20 ± 0.02	13.13 ± 0.21	10.00 ± 5.77	3733.33 ± 72.65	2822.22 ± 41.94	1705.56 ± 25.46
N08	0.36 ± 0.16	3.93 ± 0.03	31.83 ± 0.60	15.00 ± 2.89	3866.67 ± 183.33	2216.67 ± 92.80	1483.33 ± 16.67
N09	0.10 ± 0.01	0.83 ± 0.02	41.56 ± 0.54	16.67 ± 0	3405.56 ± 161.88	2566.67 ± 57.74	872.22 ± 19.24
N10	0.04 ± 0.01	2.12 ± 0.02	24.50 ± 0.44	15.56 ± 1.92	4283.33 ± 120.18	2183.33 ± 44.10	1244.44 ± 9.62
N11	0.08 ± 0.02	1.04 ± 0.02	26.50 ± 0.67	15.00 ± 2.89	3783.33 ± 44.10	2400.00 ± 100.00	733.33 ± 16.67
Ref	0.10	0.97	47.33	33.33	12633.33	2800.00	1333.33
LOD (ng/L)	0.002	0.001	0.007	0.003	0.021	0.005	0.039

**Table 6:** Predicting results obtained by SVM.

Data type	Sensitivity	Specificity	Accuracy
Isotope data	0.9394 (31/33)	0.9091 (10/11)	0.9318 (41/44)
Metallic element data	0.8788 (29/33)	0.4545 (5/11)	0.7727 (34/44)
Coupled data	0.9697 (32/33)	1 (11/11)	0.9773 (43/44)

**Table 7:** Rank results of isotope and metallic element data.

Isotopes		Metallic element			
Ranking	Variable	Ranking	Variable	Ranking	Variable
1	*δ* ^2^H	1	Cs	8	Cr
2	*δ* ^18^O	2	Cu	9	Ni
3	*δ* ^15^N	3	Ca	10	Zn
4	*δ* ^13^C	4	Rb	11	Ti
		5	Sr	12	Mg
		6	Ba	13	Mn
		7	Cd	14	Co
